# Fungal Endophytic Community Associated with Guarana (*Paullinia cupana* Var. Sorbilis): Diversity Driver by Genotypes in the Centre of Origin

**DOI:** 10.3390/jof6030123

**Published:** 2020-07-31

**Authors:** Carla Santos, Blenda Naara Santos da Silva, Ana Francisca Tibúrcia Amorim Ferreira e Ferreira, Cledir Santos, Nelson Lima, Jânia Lília da Silva Bentes

**Affiliations:** 1 CEB-Centre of Biological Engineering, Micoteca da Universidade do Minho, University of Minho, 4710-057 Braga, Portugal; carla.santos@ceb.uminho.pt (C.S.); nelson@ie.uminho.pt (N.L.); 2 Postgraduate Program in Tropical Agronomy, Federal University of Amazonas, Manaus-AM 69067-005, Brazil; blenda.naara@gmail.com (B.N.S.d.S.); ana.tiburcia@gmail.com (A.F.T.A.F.eF.); jlbentes@ufam.edu.br (J.L.d.S.B.); 3 Department of Chemical Sciences and Natural Resources, BIOREN-UFRO, Universidad de La Frontera, Temuco 4811-230, Chile

**Keywords:** mycobiota, composition, diversity, genotypes, plant organs, geographical location

## Abstract

Guarana plant is a native of the Amazon region. Due to its high amount of caffeine and tannins, the seed has medicinal and stimulating properties. The guarana industry has grown exponentially in recent years; however, little information is available about associated mycobiota, particularly endophytic fungi. The present study aimed to compare the distribution and diversity of endophytic fungi associated with the leaves and seeds of anthracnose-resistant and susceptible guarana plants produced in Maués and Manaus, Amazonas State, Brazil. A total of 7514 endophytic fungi were isolated on Potato Dextrose Agar, Sabouraud and Czapek media, and grouped into 77 morphological groups. Overall, fungal communities in guarana leaves and seeds were mainly composed by *Colletotrichum* and *Fusarium* genera, but also by *Chondrostereum*, *Clonostachys*, *Curvularia*, *Hypomontagnella*, *Lentinus*, *Neopestalotiopsis*, *Nigrospora*, *Peroneutypa*, *Phyllosticta*, *Simplicillium* and *Tinctoporellus*. Obtained results indicate that some members of *Colletotrichum* and *Fusarium* genera may have experienced dysbiosis during the guarana domestication process, suggesting that some individuals may behave as latent pathogens. The susceptible guarana genotype cultivated in Manaus presented higher fungal diversity. The relative abundance of taxa and diversity among samples suggests that communities are structured by genotype and geographic location. This is the first report of mycobiota in both guarana leaves and seeds.

## 1. Introduction

The guarana plant (*Paullinia cupana* var. *sorbilis* Mart. Ducke) is an Amazonian species with a center of origin in Maués city, of Amazon State-Brazil [1]. The seeds, the commercially exploited plant part, are characterized by high amounts of caffeine—about two to five times greater than the content found in coffee (*Coffea arabica* L.), yerba mate (*Illex paraguariensis* A. St.-Hil.) and green tea (*Camellia sinensis* L. Kuntze) [2].

The indigenous *Sateré Maué* tribe associated the use of guarana seeds with strength, vitality and disease prevention [3]. Its consumption results in changes in the nervous system [4], improvement of physical performance [5], and increased cognitive response [6]. In addition, previous studies suggest it can have protective effects against neuropathologies [7,8,9], reduction in cardiovascular diseases [10], weight loss in humans [11,12,13,14], and changes in intestinal microbiota [13]. Due to its antioxidative action, products derived from guarana can replace synthetic food antioxidants and are used in the manufacture of various cosmetics [15,16]. It also has antimutagenic, anticarcinogenic and antiallergenic properties [17,18]. These stimulating, therapeutic and medicinal properties of guarana are related to its chemical substances, such as tannins, xanthines, and especially caffeine (1,3,7-trimetilxantina) [19]. The compounds, theobromine (3,7-dimethylxanthine), theophylline (1,3-dimethylxanthine) and tannins represent 0.3%, 0.3% and 14% of the plant content, respectively [20,21].

The properties of guarana seeds make their characteristics highly requested by different industries. Currently, Brazil is the main guarana producer in the world. Most of the production is consumed in the domestic market by the carbonated beverages sector (45%), and remainder amount is used in the manufacture of syrups, powders and pharma compounds in general [22]. The growing demand for different healthy products among consumers, especially in the beverage sector, is expected to increase the guarana market to USD 8.30 billion by 2021, representing an increase of 142% over the value published in 2018, reflecting an increase of USD 5.86 billion [23].

Seeking to meet market demand, guarana industry has grown exponentially in Brazil. Currently, guarana cultivation occupies 15 thousand hectares, distributed mostly in the Brazilian States of Bahia (6500 ha) and Amazonas (8133 ha) [24]. Until 2016, the highest production per hectare was recorded in Bahia. According to a previous study published by the Brazilian Institute of Geography and Statistics (IBGE, Brazil), in the period from 2017 to 2018, a significant increase in the guarana production in the State of Rondônia, Brazil, was observed. It reached 705.8 ton.ha^−1^, leaving Amazonas in third place in terms of productivity, with 501.5 ton.ha^−1^, while Bahia State remained in the first place, with a production of 862.1 ton.ha^−1^ [25].

In the Amazon, the tropical climate, characterized as hot and humid (annual average: 27.2 °C and 2101 mm [26]), favors the establishment of insects and pathogens that significantly affect plants. The trips (*Pseudophilothrips adisi* Zur Strassen), anthracnose (*Colletotrichum guaranicola* Albuq.) and oversprouting (*Fusarium decemcellulare* Brick) are the main factors related to the stagnation of guarana production in the Amazon region [27,28,29,30]. Application of agrochemicals, cultural practices and insertion of tolerant genotypes have been used to control these problems [28,31,32].

Recent studies have demonstrated the importance of microorganisms associated to plants as biological source of new molecules and bioactive compounds. Such close relationships between hosts and their associated communities of microorganisms (or microbiota) have led to the description of the “holobiont” concept. This has been around since the early 20th century [33] but it is mostly associated with the studies of Margulis, particularly [34]. More recently the term “hologenome” was proposed—it corresponds to the entire metagenome of a holobiont, that is, the combined gene pool of the host and its microbiota [35]. In this theory, the relationship between host and its microbiota is a key aspect affecting the holobiont fitness to its environment [35]. Among the microorganisms associated to plants, endophytic fungi stand out for their multiple interactions with the host and are a good choice for exploitation of such kind of new molecules with biological activity against insects and pathogens. During plant–fungus mutual interaction, endophytes decrease attacks by herbivore and plant pathogens, favoring greater protection of the plant and production of vegetal biomass. In return, plants provide essential nutrients to endophytic fungi, and produce hormones and amino acids that modulate mycobiota by recruiting specific taxonomic groups [36,37]. However, not always do the recruited endophyte groups result in positive effects for the host. Such microorganisms may antagonize phytopathogens, facilitate disease or have neutral effect [38,39]. Positive, neutral or negative effects depend on the environment and the different combinations between host and endophyte genotypes as well as interactions with other organisms [40,41].

The understanding of the factors guiding the microbial community of guarana is extremely important because it can elucidate how these microorganisms are structured in specific niches. In addition, the symbiotic interactions between endophytic fungi and guarana have been little explored [42,43,44]. The aim of this study was to identify the taxonomic composition of endophytes on leaves and seeds of guarana (*Paullinia cupana* var. *sorbilis*), and compare culturable fungi isolated among different guarana genotypes and geographical locations of guarana production in the Amazon region, Brazil.

## 2. Materials and Methods

### 2.1. Sampling

Healthy guarana leaves and fruits from susceptible (BRS Amazonas cultivar 300) and resistant (BRS Maués cultivar 871) genotypes to both anthracnose and oversprouting diseases were collected during November 2014 in the experimental fields of Embrapa Amazônia Ocidental located in the municipalities of Manaus (MAO, 2°56′33″ S 59°56′07″ W) and Maués (MBZ, 3°23′55″ S 57°42′25″ W), in the state of Amazonas, Brazil. The collection was performed from 5 plants of each genotype in both municipalities, totalizing 20 plants. The collected material was labelled according to the origin and susceptibility with the following abbreviations: MAO 300 or MAO 871 (susceptible or resistant genotypes from Manaus); and MBZ 300 or MBZ 871 (susceptible or resistant genotypes from Maués). The labelled plant material was stored in paper bags, packed in ice and transported to the Laboratory of Microbiology and Plant Pathology of the Federal University of Amazonas (UFAM) in Manaus city, Brazil.

### 2.2. Fungal Endophyte Isolation

Endophytic fungi were isolated from leaves as described in [45]. Leaf fragments of 5 cm were submitted to superficial disinfestation in ethanol (70%, 1 min), NaCl (2%, 1 min), ethanol (70%, 30 s), followed by triple wash in sterilized distilled water. The edges of the disinfested fragments were eliminated, obtaining samples of 0.5 cm^2^, deposited in Petri dishes with PDA (Kasvi, São José do Pinhais, Paraná, Brazil), Sabouraud (Merck, Darmstadt, Germany) and Czapek (Difco^TM^, BD, Franklin Lakes, NJ, USA) media supplemented with chloramphenicol 250 mg·L^−1^ (Amresco^®^, Solon, OH, USA). For each culture medium, 100 fragments per plant were deposited, totaling 300 fragments per plant in the three culture media.

For the isolation of endophytic fungi from seeds, 25 seeds from each origin (MAO 300, MAO 871, MBZ 300 and MBZ 871) were used, totaling 100 seeds. The fruits were washed in running water and the aryl was removed, followed by surface disinfestation in aqueous solution of ethanol (70%, 2 min), NaClO (3%, 5 min), ethanol (70%, 30 s), followed by rinse thrice in sterilized distilled water. Five equidistant seeds were deposited in Petri’s dishes with PDA supplemented with chloramphenicol (250 mg·L^−1^).

The effectiveness of the disinfestation process was verified by deposition of 50 μL of the water used in the last wash in the same culture media used for endophytic fungi isolation. Petri dishes containing control, leaf fragments and seeds were kept at 28 °C without photoperiod.

After 24–72 h, the first fragments of hyphae grown from leaves and seeds were transferred to new Petri dishes containing PDA for isolation of fungal colonies. After fungal growth (7–10 days), the macro-morphological characteristics were observed. For fungi visualization, slides were prepared with lactophenol and cotton blue. Observation of reproductive fungal structures was carried out by using a Carl Zeiss^®^ (Oberkochen, Germany) light microscope and photographed with the AxioCAM ERc 5s camera with a 40× objective.

Endophytic fungal colonies were subsequently quantified and grouped into morphotypes/OTU’s (Operational Taxonomic Units) based on their cultural and reproductive structures characteristics [46,47,48,49]. In order to maintain genetic uniformity, monoconidial cultures were obtained from representatives of each OTU [50]. All monoconidial isolates were preserved using the Castellani method and deposited in the Chilean Culture Collection of Type Strains (WDCM 1111, http://ccct.ufro.cl/), hosted by the Universidad de La Frontera (Temuco, Chile).

### 2.3. DNA Extraction, Amplification and Sequencing

Genomic DNA was extracted from monoconidial cultures from representatives of each OUTs, using the Wizard^®^ Genomic DNA Purification Kit. DNA was quantified in 0.8% agarose gels using 50 ng lambda DNA (Promega, Madison, WI, USA) molecular weight marker, and the fragments were visualized using the Loccus Biotechnology Molecular Imaging Transilluminator. The 260/280 ratio for DNA quality and concentration was obtained using a Nanodrop^®^ 2000c spectrophotometer (Thermo Fisher Scientific, Waltham, MA, USA), and the final concentration adjusted to 30 ng.μL^−1^.

Internal transcribed spacer of ribosomal DNA (ITS) region was amplified using 0.2 µM of ITS1/ITS4 primers [51], 1× VWR Taq DNA Polymerase Master Mix with 1 mM MgCl_2_ (VWR, Radnor, Pennsylvania, PA, USA), and approximately 50 ng of template DNA in a total 50 µL reaction volume. PCR cycling conditions were: pre-denaturation at 95  °C for 5 min; followed by 35 cycles of denaturation at 95  °C for 1 min, primer annealing at 56  °C for 45 s, extension at 72  °C for 90  s; and final extension at 72  °C for 10 min, in a BioRad C-1000 thermocycler (BioRad, Hercules, CA, USA). Amplification success was verified in 1% agarose gel and obtained amplicons were purified according to the NZYGelpure kit (NZYtech, Lisbon, Portugal) protocol. Samples were sent for Sanger sequencing to Stab Vida Lda (Madan Parque, Caparica, Portugal). Generated electropherograms were analyzed using 4Peaks (by A. Griekspoor and Tom Groothuis, nucleobytes.com). Sequences were primarily analyzed using the Blast algorithm from NCBI National Center for Biotechnology Information (www.ncbi.nlm.nih.gov).

Phylogenetic analysis was performed by multiple alignment of the obtained ITS sequences against those of different species sequences retrieved from the NCBI database (Table S1). Alignment was performed using the MUSCLE tool [52], implemented in MEGAX software (Institute of Molecular Evolutionary Genetics, The Pennsylvania State University, USA [53,54]). Poorly aligned positions and divergent regions were eliminated using the Gblocks v.0.91b online tool (Institut de Biologia Evolutiva (CSIC-UPF), Barcelona, Spain, [55]). The most suitable substitution model was determined based on the lowest Bayesian information criterion. A Maximum Likelihood (ML) tree—based on the Kimura two-parameter [56] substitution model (K2), considering non-uniformity of evolutionary rates among sites modelled using a discrete Gamma distribution (+G) with 5 rate categories, assuming that a certain fraction of sites are evolutionary invariable (+I), and 1000 bootstrap replications [57]—was constructed using MEGAX. All positions with less than 95% site coverage data were eliminated. The obtained tree was edited in iTOL v.5.6 program (biobyte solutions GmbH, Heidelberg, Germany, [58]).

### 2.4. Fungal Diversity Analysis

The analyses of taxonomic composition were performed using the relative abundance matrix of genera, later grouped in Operational Taxonomic Unit (OTUs), where each genus reflects one OTU. Plots with genera taxonomic composition were constructed using Phyloseq R [59] and ggplot [60].

Analysis of endophytic fungi diversity was calculated by the program RStudio version 1.1.463 using the relative abundance of the taxa found in the samples. The alpha diversity, which analyzes the diversity within each sample, was estimated by genotype (BRS Amazonas and BRS Maués) (*q* = 0), Shannon diversity (*q* = 1), and Simpson diversity (*q* = 2) in the range of geographic origin (Manaus and Maués), using the Hill series, which takes into account the effective number of genera, package iNEXT [61].

## 3. Results and Discussion

The analysis of diversity and taxonomic composition of the endophytic community of guarana were based on the amount of fungi recovered from healthy guarana tissue samples in different culture media, followed by the quantification and separation by morphotypes until identification through molecular data. The diversity and composition of leaf and seed endophytes from two guarana cultivars, both of which differed as to resistance/susceptibility to anthracnose and to oversprouting, considered the main diseases of this crop, were compared within a regional scale, which includes the municipalities of Manaus and Maués Brazil, the latter being considered the center of origin of guarana.

As a result of the field collection, 7441 endophytes from 6000 guarana leaf fragments and 73 fungi obtained from 100 seeds were isolated, distributed in two different cultivars and municipalities according to Table 1. Leaf and seed fungi were grouped into 77 morphological groups. Such isolation effort allows the capture of several diversity levels of the endophytic community associated with guarana and it provides valuable resources for future studies, either in fungal taxonomy or biotechnological applications.

The amount of 77 morphotypes corresponded to 26 OTUs which were detected through molecular sequencing. Initially, the morphotypes were separated according to cultural characteristics that justified the groups’ distinction. However, different morphological groups were later identified within the same genus. Similar results were observed in the study developed by Singh et al. [62], with the endophytic fungi of *Tectona grandis*. The authors recovered 5089 isolates attributed to 45 distinct morphotypes, identified based on the ITS region in just over 23 genera. Tan et al. [63] isolated 224 endophytic fungi from various plant tissues of *Dysosma versipellis*, classified within 53 morphotypes and identified on the basis of ITS in 29 different genera. Guo et al. [64] grouped an enormous amount of endophytes of *Leptocanna chinensis* into 19 morphotypes. After sequencing the ITS region, the endophytes were collected in only 3 genera: *Diaporthe*, *Mycosphaerella* and *Xylaria*. On the other hand, Wang et al. [65] obtained a better approximation of the morphotype-taxon relation, the authors grouped the endophytic fungi into 77 morphotypes, which were divided into 64 taxa based on the analysis of the ITS sequencing. Most studies with cultivable fungi report similar results to ours, that is, higher number of morphotypes and fewer genera. Nevertheless, it is important that the morphotypes are separated, as reported in a review of traditional and molecular techniques used in studies of endophytic fungi diversity [66]. According to the authors, the arrangement within the different morphotypes does not reflect the actual phylogeny of the taxa, but it is necessary because it assists in the separation and optimization of molecular identification when one has a huge amount of individuals.

**Figure 1:**
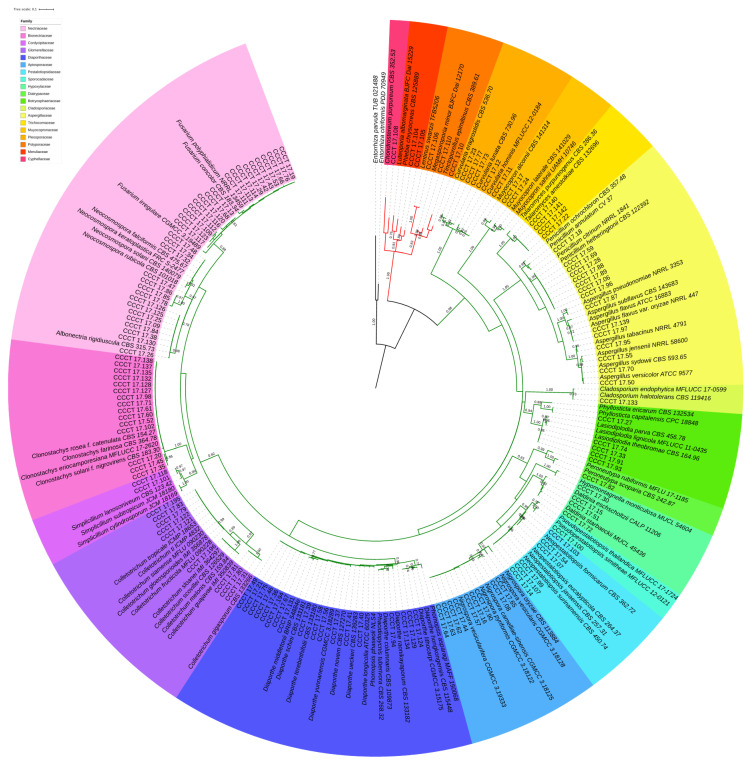
Phylogenetic tree for ITS sequence data of the 136 strains isolated from guarana with other species detailed in Table S1. *Entorrhiza parvula* TUB 021488^T^ and *E. citriformis* PDD 70949^T^ were used as outgroup. The clade highlighted by red branches is composed by *Basidiomycota* species. The clade highlighted by green branches is composed by *Ascomycota* species. Selected model: K2+G+I. The percentage of trees in which the associated taxa clusters together in the bootstrap test (1000 replicates) is shown above the branches. The tree is drawn to scale with branch lengths measured in the number of substitutions per site. All positions with less than 95% site coverage were eliminated. There were a total of 282 positions in the final dataset.

About 2486 fungi distributed in 16 morphotypes did not have their representatives identified molecularly, among them members of *Gilmaniela*, *Pithium*, *Phoma* and *Stemphillium*. These representatives needed the molecular analyses for effective identification, so they were demarcated as unidentified. While morphology can be of great value for the differentiation of some genera, some morphological characteristics of fungi may cause confusion in people not specialized in a particular genus. For example, conidiophore structure is a very helpful morphological characteristic to differentiate between *Aspergillus* and *Penicillium*. On the other hand, high levels of interspecific differences in conidial dimensions, septation and shape of aerial and sporodochial conidia in *Neocosmospora* hinder the morphological differentiation of this genus from *Fusarium* [67]. In addition, even renowned mycologists can commit faults when performing fungal identification only on the basis of morphology, which is subject to plasticity and changes caused by biotic and abiotic factors. Such endophytes lost the ability to grow in synthetic culture medium after the storage period required to process all the 7514 isolates recovered, approximately 3 to 4 months, leading to the diversity loss that these individuals could represent. The preservation method used in this study was based on nutrient reduction, suitable for various fungi, as demonstrated in [68], which maintained 44 viable taxa preserved in distilled water for one year, and in [69], who used the same method to maintain 151 basidiomycetes species for variable periods for up to 7 years. However, preservation in distilled water can cause the death of the fungus due to the absorption of water by osmosis [70]. Possibly, this happened with some of our isolates. For example, members of *Guignardia* were initially quantified and separated into a morphotype G1, but none could be retrieved for DNA extraction and sequencing. Only a fungal isolate could be recovered and sequenced within morphotype G2, later identified as the teleomorph *Phylosticta* (CCCT 17.27, see Figure 1). Many fungal isolates are lost annually because of the specificities necessary for the storage of certain species, thus, in order to preserve individuals for future studies, endophytes with sequenced DNA (Table S1) were deposited in the Colección Chilena de Cultivos Tipo (CCCT).

The initial morphological identification allowed the separation of genera that produced sexual structures, such as *Aspergillus*, *Colletotrichum*, *Fusarium* and *Penicillium*. However, a high number of fungi was classified as *Mycelia sterilia*, among them the endophytes classified molecularly as *Chondrostereum, Diaporthe*, *Hypomontagnella, Lasiodiplodia*, *Lentinus, Muyocopron*, *Peroneutypa*, *Phomopsis*, *Phylosticta*, Polyporales and *Tinctoporellus*. Some isolates did not produce conidia, and others, such as members of *Nigrospora*, lost that ability after the successive cultivation steps and under the analyzed conditions. This inability to produce conidia under laboratory conditions indicates that growth was metabolically unfavorable for the formation of reproductive structures [66]. Although conidiophores and conidia represent commonly reported characteristics in the literature, their value can be limited, since more than 50% of the total endophytic fungi usually does not sporulate on the used substrates [71,72]. Molecular biology analysis through amplification of the ITS region allowed the identification to the genus level, especially of sterile fungi that could not be classified in any taxonomic setting based on the morphology. The ITS region, known as the fungal barcode, is highly polymorphic, easily amplified, with genetic information that allows intraspecific and interspecific distinction of many members of the phyla *Ascomycota* and *Basidiomycota*, with reliable taxonomic classification at the genus level for most fungi [73]. Based on the molecular data (Figure 1), it was possible to confirm the identification of *Aspergillus*, *Colletotrichum*, *Cladosporium*, *Curvularia*, *Fusarium*, *Neopestalotiopsis* and *Penicillium*, and other lesser common fungi in the laboratory routine, such as *Chondrostereum*, *Muyocopron*, *Peroneutypa* and *Tinctoporellus*.

### 3.1. Culture Medium

**Figure 2:**
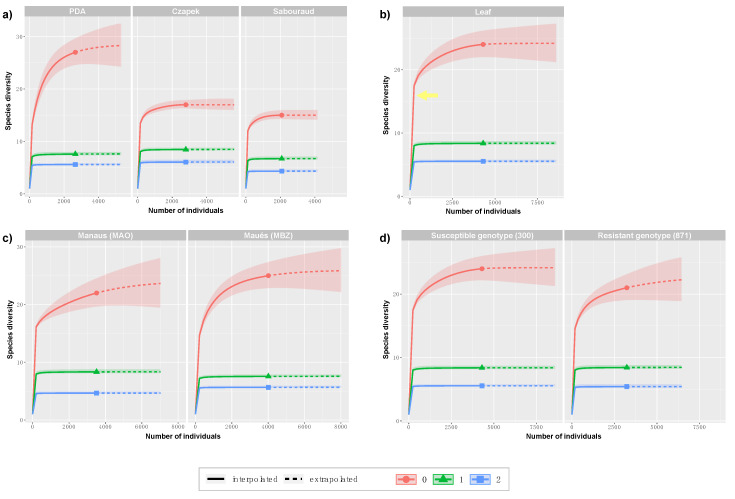
Rarefaction and extrapolation curves of species alpha diversity (*q* = 0), Shannon diversity (*q* = 1), and Simpson diversity (*q* = 2) for the following comparisons: (**a**) used culture media (PDA, Czapek and Sabouraud); (**b**) plant tissue used for sampling (as no rarefaction was achieved for seeds, the yellow arrow indicates the maximum q = 0 value estimated for seeds); (**c**) municipalities of Manaus (MAO) and Maués (MBZ) where guarana samples were collected; and (**d**) susceptible (BRS300) and resistant (BRS871) guarana genotype.

Fungi isolated from guarana in different culture media resulted in the highest number of endophytes in Czapek culture medium (2791 isolates), followed by PDA (2643) and Sabouraud (2080). The number of genera obtained varied according to the culture medium used. The largest number of unique OTUs, that is, genera exclusively found in a certain culture medium, was found in PDA (*Hypomontagnella*, *Muyocopron*, *Phyllosticta*, *Pseudopestalotiopsis* and *Talaromyces*). The Czapek and Sabouraud media provided only two unique OTUs obtained in these media (*Peroneutypa* and *Tinctoporellus*). Three OTUs (*Colletotrichum*, *Fusarium* and *Penicillium*) were obtained from the four samples (MAO 300, MAO 871, MBZ 300 and MBZ 871) in the three different culture media used. The largest number of unique OTUs was isolated in PDA medium in the city of Maués (4), and in the genotype BRS300 (5). The diversity parameters analyzed for the culture media (Figure 2a) show that PDA has greater sample richness (*q* = 0) when compared to Czapek and Sabouraud. However, the parameters *q* = 1 and 2 indicated that the three culture media did not differed in their capacity to capture the diversity present in the guarana samples.

Our results show that isolation of endophytic fungi translated in the higher number of isolates in Czapek and greater diversity in PDA medium. These results differ from those found in [44], where the authors had better results regarding the incidence and diversity of guarana endophytes in Manioc Dextrose Agar (MDA) when compared to PDA medium. The culture media reported in our study did not differed in the diversity measures considered more reliable (*q* ≥ 1) [74]. However, PDA provided five genera exclusively obtained in this culture medium, possibly due to the favorable conditions of this medium in the development of most filamentous fungi [75].

### 3.2. Taxonomic Composition

**Figure 3:**
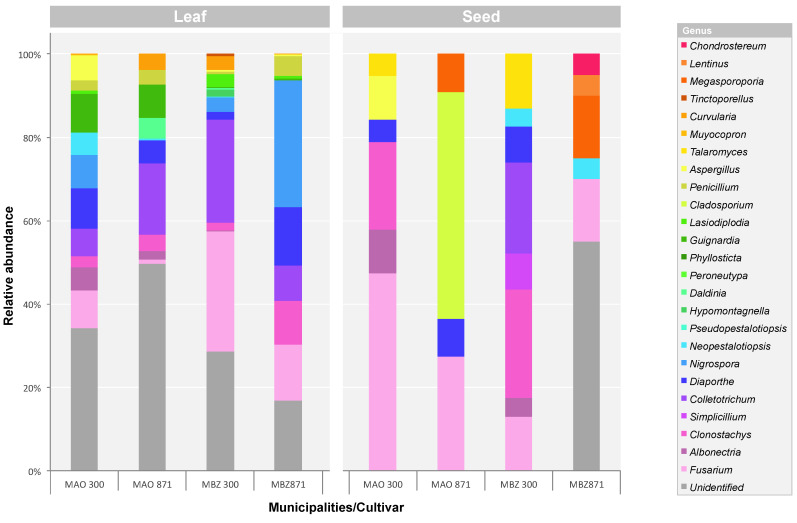
Genus level taxonomic composition of the fungal isolates obtained from guarana leaves and seeds of susceptible (BRS300) and resistant (BRS871) plant genotypes from Manaus (MAO) and Maués (MBZ), Amazonas, Brazil.

The fungal community of guarana isolated here is mainly composed by members of *Ascomycota*, phylum that also prevails in other vegetal species [76,77], with rare exceptions such as *Hevea*, where most of the endophytes obtained in [78] are basidiomycetes. About 67% (5005) of the endophytic isolates obtained from guarana are inserted in the Ascomycotina, with members of the classes *Sordariomycetes* (55%), *Dothideomycetes* (8%) and *Eurotiomycetes* (4%). Basidiomycotina group is only represented by 0.3% (23) of the isolates, with members exclusively belonging to *Agaricomycetes*. The families with the greatest relative abundance (RA) were *Nectriaceae* (17%), *Glomerellaceae* (16%), *Apiosporaceae* (8%) and *Diaporthaceae* (6%). In total, 19 families and 25 genera were identified, as shown in Figure 1 and Figure 3.

In general, the taxonomic composition of guarana had two dominant OTUs, that is, with high RA, *Colletotrichum* (16%) and *Fusarium* (15%). Another 12 OTUs had RA ranging from 1 to 7.9%, and were considered as frequent, typical or common, making up half of the identified guarana isolates (52.4%). The remaining 11 OTUs with AR < 1% were considered rare. The isolation of rare endophytic fungi such as *Cladosporium*, *Lentinus*, *Simplicillium*, *Pseudopestalotiopsis*, *Talaromyces* and *Tinctoporellus* suggests that the isolation and sampling procedures were appropriately employed. In general, the endophytes isolated from guarana leaves and seeds represent little explored niches, and resulted in the first report of 11 genera in endophytic communities of *P. cupana*: *Clonostachys*, *Curvularia*, *Chondrostereum*, *Hypomontagnella*, *Lentinus*, *Neopestalotiopsis*, *Nigrospora*, *Peroneutypa, Phyllosticta*, *Simplicillium* and *Tinctoporellus*. These genera, reported here for the first time in guarana, have already been cited in studies on the biological control of pests and diseases in cultivated plants. For example, strong fungicidal activity was evidenced by an extract produced from *Lentinus crinitus,* capable of inhibiting more than 92% of the conidial spores of *Fusarium* sp. [79]. The fungus *Clonostachys rosea* is considered an effective organism: entomopathogenic, mycoparasitic and nematophagus. Such capacities are associated with, among other factors, the production of serine protease, an enzyme with important role during biological control [80,81,82]. The strains *C. rosea* MpA/MpB and *Bionectria* sp. 6.21 reported in [83,84] have antagonistic activity against phytopathogens through mycoparasitism and the production of secondary metabolites that aid in the breakdown and degradation of the cell wall. Another mycoparasite species, also found in guarana, is *Simplicillium lanosoniveum* S-599 which parasites fungi by secreting proteases [85].

Most of the taxa associated with guarana were previously reported in the literature as having beneficial ecological functions, like plant growth-promoting fungi (PGPF) which have the natural ability to stimulate seedling vigor, seed germination rate, root morphogenesis and development, shoot growth, yield, flowering, plant composition and photosynthetic efficiency [86,87,88,89,90,91]. This ability can occur through one or more mechanisms such as production of volatile organic compounds (VOC’s) and phytohormones, antagonism to phytopathogens, amelioration of abiotic stresses and enhanced nutrient availability [92]. For instance, Naraghi et al. [93] found that the endophyte *Talaromyces flavus* (TF-Po-V-50 and TF-Co-M-23) can promote the development and increase the biomass of cotton and potato plants mediated by the seed treatment method; Hossain et al. [94] reported that *Penicillium simplicissimum* GP17-2 could induce the host-plant defense system by the activation of multiple chemical signals. The authors observed that *Arabidopsis thaliana* plants inoculated with GP17-2 presented a clear induced systemic resistance (ISR) to *Pseudomonas syringae* pv. *tomato* DC3000. Other genera have also been reported with the ability to increase plant growth such as *Aspergillus, Cladosporium*, *Clonostachys*, *Curvularia*, *Phomopsis* and *Talaromyces* [87,95,96,97,98]. In the present study, it was possible to verify that the same genera occur in the guarana endophytic community. One can hypothesize that these isolates improve guarana plant growth through different mechanisms, but further studies are needed to confirm.

### 3.3. Composition of the Endophytic Microbiota of Genotypes and Municipalities

The distribution of genera according to plant genotypes and collection municipalities is shown in Figure 3.

In the obtained leaves of MAO 300, five OTUs prevailed over the others, *Diaporthe* (10%), *Fusarium* (9%), *Guignardia* (9%), *Nigrospora* (8%) and *Colletotrichum* (7%). In MBZ 300 only *Colletotrichum* (25%) and *Fusarium* (29%), formed the group of predominant individuals. In the genotype BRS871 from Maués the genera *Nigrospora* (30%), *Fusarium* (14%), *Diaporthe* (14%), *Clonostachys* (10%) and *Colletotrichum* (8%) showed higher RA values. A smaller number of OTUs prevailed in MAO 871, only *Colletotrichum* (17%) and *Guignardia* (8%). The foliar endophytic community is composed of 20 OTUs, where 8 OTUs were found in both genotypes and municipalities: *Clonostachys*, *Colletotrichum*, *Curvularia, Diaporthe*, *Fusarium*, *Guignardia*, *Neopestalotiopsis* and *Penicillium*. The largest number of unique OTUs, that is, those found only in a given sample, was found in MBZ 300 (4) and MAO 300 (2). In the MBZ 871 sample no unique OTU was observed and only one unique OTU, *Phyllosticta*, was obtained from MAO 871.

The OTUs obtained from seeds varied according to plant location and genotype. The endophytes from the MBZ 300 sample were mainly inserted in the genera *Colletotrichum* (22%), *Clonostachys* (26%), *Fusarium* (13%), *Talaromyces* (13%), *Diaporthe* (9%) and *Simplicillium* (9%). In MAO 300, other groups had high RA, *Fusarium* (47%), *Clonostachys* (21%), *Aspergillus* (11%) and *Albonectria* (11%). In cultivar BRS871 the most abundant OTUs were *Fusarium* (15%) and *Megasporoporia* (15%) in MBZ 871, and *Cladosporium* (55%) and *Fusarium* (27%) in MAO 871. The microbial seed community had a higher number of unique OTUs in Maués, in samples MBZ 300 (2) and MBZ 871 (2). In Manaus, MAO 300 and MAO 871, one unique OTU was obtained in each sample, *Aspergillus* and *Cladosporium*, respectively. Only one OTU (*Fusarium*) was isolated in all guarana genotypes and municipalities studied. In the susceptible cultivar (BRS300), the highest total amount of OTUs, 8 and 6, were present in the MBZ300 and MAO300 samples, respectively.

The mycobiota present in guarana plants is heterogeneous, varying in distribution and abundance of genera according to plant genotypes and municipalities of sample collection. The present results suggest that structuring of guarana fungal community (cultivable organisms) is directed both by the genetics of the host plant as well as by the geographic location, especially in leaves. These results are in line with previous studies of grapevines [99] and tomato [100] plants that have shown that different plant organs, genotypes of the same plant species and even sampling positions in the farmland can harbor partially different microbiomes. Guarana had high dominance of *Colletotrichum* and *Fusarium*, known as well established organisms, with different life style types [98]. Differences in life style depend on environmental conditions, fungal species, host and its maturity. *Colletotrichum* species life styles, for example, can be broadly categorized as latent or quiescent, endophytic, hemibiotrophic and necrotrophic [101,102,103]. Both genera have been extensively associated with the endophytic community in different plants [62,104,105] but are also important pathogens of a wide range of hosts such as pepper, soy, alfalfa and many other cultivable plants [101,106,107]. Several studies have shown that pathogenic or parasitic fungi are found as endophytes [108,109,110,111,112]. In this situation, endophytes are latent pathogens that infect the plant, and persist in a dormant phase without causing symptoms in the host [113]. The symptoms and signs of the disease appear rapidly in response to physiological changes of the plant, either by its stage of maturity or when subjected to nutritional and environmental changes. Abiotic or biotic stress can trigger the pathogenic activity of endophytes when the host is not able to limit fungal growth [101,109,114]. In leaves, both genera were observed with high RA in Maués, origin of the dispersion of guarana, and in BRS300, cultivar susceptible to anthracnose and to supersprouting. In seeds, members of *Fusarium*, were dominant in all samples, regardless of genotype and locality. On the other hand, individuals from *Colletotrichum* were only obtained from seeds of MBZ 300. This suggests that *Fusarium* endophytes possibly suffer vertical transmission in guarana, passing successively through generations and increasing their presence in the next generation of seedlings [115,116]. On the other hand, *Colletotrichum* endophytes are most probably acquired via horizontal transmission, therefore being influenced by environment and geographical location.

Anthracnose and oversprouting are between the main diseases affecting guarana crops. The first mainly affects aerial organs by severe tissue necrosis and the latter is characterized by malformed tissue and organs in the nodes or branching points. Both diseases lead to guarana plant decline, affecting plant growth and flowering, and to a reduction in crop productivity. *Colletotrichum* and *Fusarium* endophytes are closely related with pathogens of anthracnose and oversprouting, and some members may have undergone modifications related to the ecological pressure suffered with the establishment of guarana monocultures in Amazonas and favorable climatic conditions, such as humidity and temperature extremes suitable for the multiplication of microorganisms. The period in which the process of endophyte-pathogen modification occurred, that is, the product of the co-evolutionary process, is an extremely short geological time, as demonstrated in [117]. The author suggested a recent domestication of guarana populations and dispersion originating from Maués just over 600 years ago. The process of domestication, which relied in monoclonal cultures of guarana from Maués and expansion throughout the Amazonas region [19], possibly caused “dysbiosis”, that is, an imbalance in the microbial communities generating the transition of some members to a pathogenic phase, a process already observed in *Colletotrichum magna* [118], *Fusarium graminearum* [119] and *Lasiodiplodia* sp. [120]. These results suggest that *Colletotrichum* and *Fusarium* could represent potential pathogens. However, the endophytic microbiota that makes guarana also includes other genera such as *Aspergillus*, *Clonostachys*, *Nigrospora*, *Phomopsis (Diaporthe)* and *Talaromyces*, previously cited in the literature as having a role in plant bioprotection. In this way, future studies could verify how these endophytes influence the guarana plant in order to elucidate the types of interactions that occur, positive, negative or neutral, and whether such interactions can be manipulated in favor of increased production and protection of guarana.

### 3.4. Diversity Analysis

**Figure 4:**
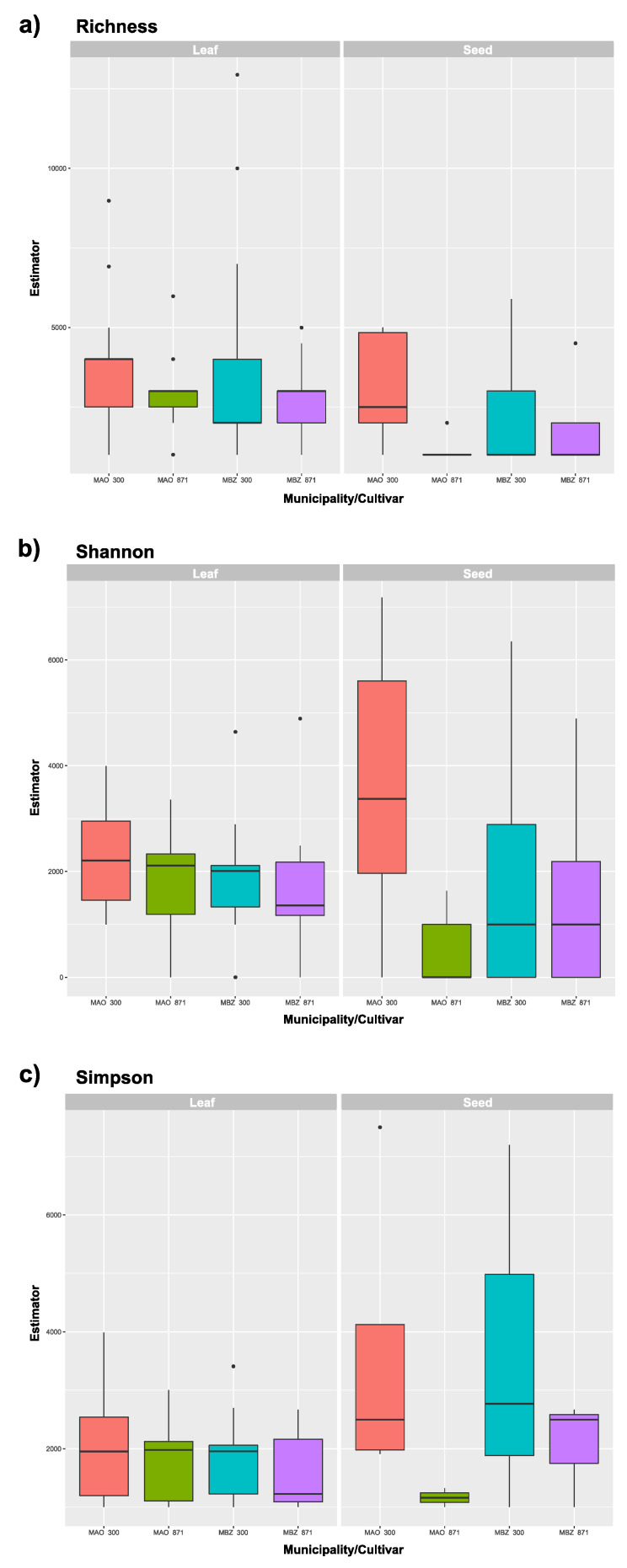
Diversity indexes estimates based on fungal populations obtained from guarana leaves and seeds of susceptible (BRS300) and resistant (BRS871) plant genotypes from Manaus (MAO) and Maués (MBZ), Amazonas, Brazil. (**a**) species richness; (**b**) Shannon diversity; and (**c**) Simpson diversity.

Several rarefaction and extrapolation curves of diversity measures comparisons are presented in Figure 2. First we aimed to compare diversity values of different plant organs used for fungal isolation. However, considering the very low number of individuals obtained from seeds (Table 1), no rarefaction was achieved and diversity measures were not plotted. In Figure 2b, a reference to the maximum *q* = 0 rarefaction value obtained for seeds is shown. In comparison, it is possible to see that leaves have greater richness (*q* = 0), that is, a higher number of genera. Furthermore, Maués (Figure 2c) and the susceptible guarana genotype BRS300 (Figure 2d) also have greater richness (*q* = 0) with no differences observed in the other indexes studied (*q* = 1, 2). It is interesting to note that the rarefaction curves in Figure 2b–d were estimated by the combined analysis of leaf and seed isolates. However, when analyzing them separately, the patterns of diversity change (Figure 4). The richness, Shannon and Simpson diversity indexes analyzed by BoxPlots show that the municipality of Manaus (MAO) and the susceptible genotype (BRS300) are the most diverse. In leaves, the greatest diversity was observed in samples MAO 300 and MAO 871 while the seeds samples MAO 300 and MBZ 300 had higher indices within the observed parameters. Variation in diversity estimates appears to be greater in seed samples, which can be explained by the sensitivity of the Shannon index (*q* = 1, Figure 4b) and of the Simpson index (*q* = 2, Figure 4c) to unique and abundant and only to abundant genera, respectively. The lower number of analyzed seed isolates exacerbates the observed RA values for the present OTUs, particularly in sample MAO 300, where two OTUs contain 68% of the RA.

The study of the microbial community and its diversity can be influenced by the traditional isolation method, since culture dependent methods are highly laborious and can hinder the isolation, enumeration and maintenance of viable fungus species, as well as obtaining non-culturable and biotrophic species [62,121]. Recently, diversity studies have been conducted using methods independent of microorganism cultivation, such as Next Generation Sequencing (NGS) approaches. However, these methods may produce errors and have been shown to overestimate the number of microorganisms present in the samples [66,121,122,123,124]. A previous work comparing culture-based endophyte diversity data with NGS data from the same host plant revealed that the culture-dependent method by itself has the ability to reveal a real qualitative picture of fungal endophytes [123,125]. In addition, the traditional culture method is the only way to isolate microorganisms for future studies in the laboratory in order to explore the production of molecules that may be useful for various purposes.

In guarana, the diversity between geographical locations varied according to the source plant material, possibly related to the more robust amount of fungi sampled in leaves, and genotype, with the susceptible genotype having higher diversity indexes. This suggests that the microbial diversity of guarana can be influenced by both genotype and geographical location. Our results are consistent with previous studies, such as [126], where the authors demonstrated that even in the face of disturbances such as the application of fungicides and presence of pests, the most determinant factors of the endophytic community of *Ageratina altissima* were the locality and the cultivar. In another study that analyzed the abundance, diversity, species composition and relative affinity with the host of two tree species, the community of endophytes differed according to the locality and host species [127]. Similar results were observed in the endophytic microbiota of *Elymus mollis*, *Ammophila arenaria* and *Ammophila breviligulata*, differing from the soil microbial community, which was strictly influenced by environmental factors, not by the cultivar or location [128].

In the present study, the susceptible genotype and, in general, the municipality of Manaus, were shown to have greater diversity. Similar results were observed in the seeds and roots of guarana studied in [42]. The authors related the richness of the susceptible genotype to the host vulnerability to microbial infections. They also reported greater diversity in Manaus, correlated with the large amount of inoculum that the planting received because it is located near an urban area, different from the one found in Maués, located in a rural area. The genotypes covered in this study have some similar characteristics. Both were originally selected from progenies located in the municipality of Maués, with clonal propagation by rooting of cuttings, average annual seed yield (1.49 kg·plant^−1^ and 1.55 kg·plant^−1^) and similar caffeine contents (3.92% and 4.04%) [129,130]. However, they differ in adapting to different conditions. Interestingly, the susceptible genotype observed in this study to have greater endophyte diversity was reported in other studies to have better rooting of cuttings [129], lower mortality rate [131] and higher yield of production (kg/plant) even with increased competition for nutrients (plant/area) [132]. More recent works have shown that plant microbiota plays a key role in host adaptation. The dynamic genetic change of the microorganisms provides the necessary time for the host adaptation to the adverse conditions, improving plant adjustment and survival [133]. Considering the idea that the microbial community possibly exerts influence on the performance and adaptation of the studied guarana plants, achieving a better understanding of how host genetic variation and geographic location affects the microbial community should be pursued in future efforts to incorporate biology into evolutionary ecology and agricultural science.

## 4. Conclusions

Guarana cultivable mycobiota is heterogeneous varying in distribution and abundance of genera according to host plant genotype and geographic location. It is formed by 25 genera of endophytic fungi, including the highly abundant *Colletotrichum* and *Fusarium*. Culture-dependent methods such as the strategy adopted in this study give, by themselves, accurate qualitative pictures of fungal diversity present in the host plant. Nevertheless, improvement of endophyte preservation and identification techniques is necessary. In the present study, several isolates representing 16 morphotypes were left unidentified since they have lost their ability to grow in synthetic medium and could not be identified based on molecular techniques, which lead to the potential diversity loss that these individuals could represent.

The diversity was higher in the BRS300 susceptible cultivar and in the municipality of Manaus. The main drivers of microbial community composition and diversity in guarana are plant genotype and geographic location, as evidenced by the difference of dominant endophytes in the sampling units, presence of large number of OTUs found exclusively in certain samples, and diversity patterns. This is in accordance with previous studies that have related susceptible genotype richness to host vulnerability to microbial infections and the greater diversity in Manaus with large amount of inoculum received by the planting.

*Colletotrichum* and *Fusarium* are known pathogens responsible, respectively, for anthracnose and oversprouting diseases that compromise plant health and reduce crop productivity. In this study, they were isolated as endophytes from healthy guarana tissue, possibly representing potential pathogens. However, the endophytic community of guarana includes other fungal genera previously associated with plant bioprotection.

Future studies focusing on how host genetic variation and geographic location affect the microbial community present in guarana; what types of interactions between endophytes and with the host; and whether such interactions can be manipulated to improve the fitness of the holobiont are of great interest in order to increase guarana plant protection and production.

## Figures and Tables

**Table 1:** Number of endophytic isolates from guarana plants (*Paullinia cupana* var. *sorbilis*), from the cultivars susceptible BRS300 and resistant BRS871, collected in the municipalities of Manaus and Maués AM, Brazil.

		Leaves		Seeds		
	Cultivar	BRS300	BRS871	BRS300	BRS871	Total
**Local**	Manaus	1533	1947	19	11	3510
	Maués	2721	1240	23	20	4004
	**Total**	4254	3187	42	31	7514

## Supplementary Materials

The following are available online at https://www.mdpi.com/2309-608X/6/3/123/s1, Table S1: Strains used for phylogenetic analysis. A list of GenBank ITS accession numbers for a set of selected species and for the 136 guarana isolates amplified here is given.

## References

[1] Atroch A.L., Nascimento Filho F.J., Rodrigues S., Silva E.O., Brito E.S., Rodrigues S., Silva E.O., Brito E.S. (2018). Guarana—*Paullinia cupana* Kunth var. *sorbilis* (Mart.) Ducke. Exotic Fruits.

[2] Babu K.M., Church R.J., Lewander W. (2008). Energy drinks: The new eye-opener for adolescents. Clin. Pediatr. Emerg. Med..

[3] Lorenz S.D.S. (1992). Sateré-Mawé: Os Filhos do Guaraná.

[4] Higgins J.P., Tuttle T.D., Higgins C.L. (2010). Energy beverages: Content and safety. Mayo Clin. Proc..

[5] Smith N., Atroch A.L. (2010). Guaraná’s journey from regional tonic to aphrodisiac and global energy drink. Evid. Based Complement. Alt. Med. eCAM.

[6] Pomportes L., Davranche K., Brisswalter I., Hays A., Brisswalter J., Pomportes L., Davranche K., Brisswalter I., Hays A., Brisswalter J. (2014). Heart rate variability and cognitive function following a multi-vitamin and mineral supplementation with added guarana (*Paullinia cupana*). Nutrients.

[7] Bittencourt L.d.S., Zeidán-Chuliá F., Yatsu F.K.J., Schnorr C.E., Moresco K.S., Kolling E.A., Gelain D.P., Bassani V.L., Moreira J.C.F. (2014). Guarana (*Paullinia cupana* Mart.) prevents β-amyloid aggregation, generation of advanced glycation-end products (AGEs), and acrolein-induced cytotoxicity on human neuronal-like cells. Phytother. Res..

[8] Boasquívis P.F., Silva G.M.M., Paiva F.A., Cavalcanti R.M., Nunez C.V., de Paula Oliveira R. (2018). Guarana (*Paullinia cupana*) extract protects *Caenorhabditis elegans* models for Alzheimer disease and Huntington disease through activation of antioxidant and protein degradation pathways. Oxid. Med. Cell. Longev..

[9] De Oliveira D.M., Barreto G., Galeano P., Romero J.I., Holubiec M.I., Badorrey M.S., Capani F., Giraldez Alvarez L.D. (2011). Paullinia cupana Mart. var. *sorbilis* protects human dopaminergic neuroblastoma SH-SY5Y cell line against rotenone-induced cytotoxicity. Hum. Exp. Toxicol..

[10] Ruchel J.B., Rezer J.F.P., Thorstenberg M.L., dos Santos C.B., Cabral F.L., Lopes S.T.A., da Silva C.B., Machado A.K., da Cruz I.B.M., Schetinger M.R.C. (2016). Hypercholesterolemia and ecto-enzymes of purinergic system: Effects of *Paullinia cupana*. Phytother. Res..

[11] Bortolin R.C., Vargas A.R., Ramos V.D.M., Gasparotto J., Chaves P.R., Schnorr C.E., da Boit Martinello K., Silveira A.K., Gomes H.M., Rabelo T.K. (2019). Guarana supplementation attenuated obesity, insulin resistance, and adipokines dysregulation induced by a standardized human western diet via brown adipose tissue activation. Phytother. Res..

[12] Lima N.D.S., Teixeira L., Gambero A., Ribeiro M.L. (2018). Guarana (*Paullinia cupana*) stimulates mitochondrial biogenesis in mice fed high-fat diet. Nutrients.

[13] Martel J., Ojcius D.M., Chang C.-J., Lin C.-S., Lu C.-C., Ko Y.-F., Tseng S.-F., Lai H.-C., Young J.D. (2017). Anti-obesogenic and antidiabetic effects of plants and mushrooms. Nat. Rev. Endocrinol..

[14] Santana Á.L., Macedo G.A. (2018). Health and technological aspects of methylxanthines and polyphenols from guarana: A review. J. Funct. Foods.

[15] Basile A., Ferrara L., Pezzo D.M., Mele G., Sorbo S., Bassi P., Montesano D. (2005). Antibacterial and antioxidant activities of ethanol extract from *Paullinia cupana* Mart. J. Ethnopharmacol..

[16] Hamerski L., Vieira Somner G., Tamaio N. (2013). *Paullinia cupana* Kunth (Sapindaceae): A review of its ethnopharmacology, phytochemistry and pharmacology. J. Med. Plants Res..

[17] Avila-Sosa R., Montero-Rodríguez A.F., Aguilar-Alonso P., Vera-López O., Lazcano-Hernández M., Morales-Medina J.C., Navarro-Cruz A.R. (2019). Antioxidant properties of amazonian fruits: A mini review of in vivo and in vitro studies. Oxid. Med. Cell. Longev..

[18] Cadoná F.C., Rosa J.L., Schneider T., Cubillos-Rojas M., Sánchez-Tena S., Azzolin V.F., Assmann C.E., Machado A.K., Ribeiro E.E., da Cruz I.B.M. (2017). Guaraná, a highly caffeinated food, presents in vitro antitumor activity in colorectal and breast cancer cell lines by inhibiting AKT/mTOR/S6K and MAPKs pathways. Nutr. Cancer.

[19] Marques L.L.M., Ferreira E.D.F., Paula D.M.N., Klein T., Mello D.J.C.P. (2018). *Paullinia cupana*: A multipurpose plant—A review. Braz. J. Pharmacogn..

[20] Antonelli-Ushirobira T.M., Kaneshima E.N., Gabriel M., Audi E.A., Marques L.C., Mello J.C.P. (2010). Acute and subchronic toxicological evaluation of the semipurified extract of seeds of guaraná (*Paullinia cupana*) in rodents. Food Chem. Toxicol..

[21] Marx F. (1990). Analysis of guarana seeds II. Studies on the composition of the tannin fraction. Z. Lebensm. Unters. Forsch..

[22] Pinto C.E.D.L., Atroch A.L., Fajardo J.D.V., Nascimento Filho D.F.J. (2018). Seleção de clones de guaranazeiro para adaptabilidade e estabilidade no estado do Amazonas. Rev. Ciênc. Agrár..

[23] Market Data Forecast (2019). Guarana Seed Extract Market by Form (Powder and Liquid) by Distribution (Health Stores, Drug Stores, Online Retailing and Other Channels) by Application (Pharmaceuticals, Dietary Supplements, Cosmetics, Food and Beverages, and Others), and by Region—Global Industry Analysis, Size, Share, Growth, Trends, And Forecast To 2024.

[24] IBGE (2017). Levantamento sistemático da produção agrícola. Intituto Brasileiro de Geografia e Estatistica.

[25] IBGE (2018). Levantamento sistemático da produção agrícola: Pesquisa mensal de previsão e acompanhamento das safras agrícolas no ano civil. Intituto Brasileiro de Geografia e Estatistica.

[26] Clima Maués: Temperatura, Tempo e Dados Climatológicos Maués—Climate-Data.org. https://pt.climate-data.org/america-do-sul/brasil/amazonas/maues-879673/.

[27] Albuquerque F.C. (1960). Antracnose do Guaraná.

[28] Araújo J.C.A., Pereira J.C.R., Gasparotto L., Arruda D.M.R. (2006). O Complexo Superbrotamento do Guaranazeiro e Seu Controle.

[29] Gonçalves J.R.C. (1967). Notas Sobre as Doenças e Pragas do Guaraná no Estado do Amazonas.

[30] Tavares A.M., Atroch A.L., Nascimento Filho D.F.J., Pereira J.C.R., Araújo D.J.C.A., Moares L.A.C., Santos L.P., Garcia M.V.B., Arruda D.M.R., Sousa N.R. (2005). Cultura do Guaranazeiro no Amazonas.

[31] Araújo J.C.A., Pereira J.C.R., Gasparotto L., Arruda D.M.R., Moreira A. (2007). Antracnose do Guaranazeiro e Seu Controle.

[32] Araújo J.C.A., Pereira J.C.R., Gasparotto L., Arruda D.M.R., Nascimento Filho D.F.J., Moreira A. Avaliação de fungicidas no controle da antracnose do guaranazeiro. Proceedings of the Embrapa Amazônia Ocidental-Artigo em Anais de Congresso (ALICE).

[33] Baedke J., Fábregas-Tejeda A., Delgado A.N. (2020). The holobiont concept before Margulis. J. Exp. Zoology Part B Mol. Dev. Evol..

[34] Margulis L., Fester R. (1991). Symbiosis as a Source of Evolutionary Innovation: Speciation and Morphogenesis.

[35] Zilber-Rosenberg I., Rosenberg E. (2008). Role of microorganisms in the evolution of animals and plants: The hologenome theory of evolution. FEMS Microbiol. Rev..

[36] Toju H., Guimarães P.R., Olesen J.M., Thompson J.N. (2014). Assembly of complex plant-fungus networks. Nat. Commun..

[37] Vandenkoornhuyse P., Quaiser A., Duhamel M., Le Van A., Dufresne A. (2015). The importance of the microbiome of the plant holobiont. New Phytol..

[38] Busby P.E., Ridout M., Newcombe G. (2016). Fungal endophytes: Modifiers of plant disease. Plant Mol. Biol..

[39] Raghavendra A.K.H., Newcombe G. (2013). The contribution of foliar endophytes to quantitative resistance to *Melampsora* rust. New Phytol..

[40] Ahlholm J.U., Helander M., Henriksson J., Metzler M., Saikkonen K. (2002). Environmental conditions and host genotype direct genetic diversity of *Venturia ditricha*, a fungal endophyte of birch trees. Evolution.

[41] Wäli P.R., Helander M., Nissinen O., Saikkonen K. (2006). Susceptibility of endophyte-infected grasses to winter pathogens (snow molds). Can. J. Bot..

[42] Azevedo Silva F., Liotti R.G., Boleti A.P.D.A., Reis É.D.M., Passos M.B.S., dos Santos E.L., Sampaio O.M., Januário A.H., Branco C.L.B., Silva D.G.F. (2018). Diversity of cultivable fungal endophytes in *Paullinia cupana* (Mart.) Ducke and bioactivity of their secondary metabolites. PLoS ONE.

[43] Elias L.M., Fortkamp D., Sartori S.B., Ferreira M.C., Gomes L.H., Azevedo J.L., Montoya V.Q., Rodrigues A., Ferreira A.G., Lira S.P. (2018). The potential of compounds isolated from *Xylaria* spp. as antifungal agents against anthracnose. Braz. J. Microbiol..

[44] Sia D.E., Marcon J., Luvizotto D., Quecine M., Tsui S., Pereira J., Pizzirani-Kleiner A., Azevedo J. (2013). Endophytic fungi from the Amazonian plant *Paullinia cupana* and from *Olea europaea* isolated using cassava as an alternative starch media source. Springer Plus.

[45] Souza D.A.Q.L., Souza D.A.D.L., Astolfi Filho S., Pinheiro M.L.B., Sarquis M.I.D.M., Pereira J.O. (2004). Atividade antimicrobiana de fungos endofíticos isolados de plantas tóxicas da Amazônia: *Palicourea longiflora* (aubl.) Rich e *Strychnos cogens* Bentham. Acta Amazon..

[46] Barnett H.L., Hunter B.B. (1972). Illustrated Genera of Imperfect Fungi.

[47] Seifert K., Morgan-Jones G., Gams W., Kendrick B. (2011). The Genera of Hyphomycetes.

[48] Pitt J.I. (1979). The Genus Penicillium and Its Teleomorphic States Eupenicillium and Talaromyces.

[49] Sutton B.C. (1980). The Coelomycetes. Fungi Imperfecti with Pycnidia, Acervuli and Stromata.

[50] Takashio M. (1974). Single-spore and single-cell cultures of fungi. Two new methods particularly useful in the isolation of fungal spores and cells. Ann. Microbiol..

[51] White T.J., Bruns T., Lee S., Taylor J., Innis M.A., Gelfand D.H., Sninsky J.J., White T.J., Innis M.A., Gelfand D.H., Sninsky J.J., White T.J. (1990). Amplification and direct sequencing of fungal ribosomal RNA genes for phylogenetics. PCR Protocls: A Guide to Methods and Applications.

[52] Edgar R.C. (2004). MUSCLE: Multiple sequence alignment with high accuracy and high throughput. Nucleic Acids Res..

[53] Kumar S., Stecher G., Li M., Knyaz C., Tamura K. (2018). MEGA X: Molecular Evolutionary Genetics Analysis across Computing Platforms. Mol. Biol. Evol..

[54] Stecher G., Tamura K., Kumar S. (2020). Molecular Evolutionary Genetics Analysis (MEGA) for macOS. Mol. Biol. Evol..

[55] Castresana J. (2000). Selection of conserved blocks from multiple alignments for their use in phylogenetic analysis. Mol. Biol. Evol..

[56] Kimura M. (1980). A simple method for estimating evolutionary rates of base substitutions through comparative studies of nucleotide sequences. J. Mol. Evol..

[57] Felsenstein J. (1985). Confidence limits on phylogenies: An approach using the bootstrap. Evolution.

[58] Letunic I., Bork P. (2019). Interactive tree of life (iTOL) v4: Recent updates and new developments. Nucleic Acids Res..

[59] McMurdie P.J., Holmes S. (2013). Phyloseq: An R package for reproducible interactive analysis and graphics of microbiome census data. PLoS ONE.

[60] Wickham H. (2009). ggplot2: Elegant Graphics for Data Analysis.

[61] Hsieh T.C., Ma K.H., Chao A. (2016). iNEXT: An R package for rarefaction and extrapolation of species diversity (Hill numbers). Methods Ecol. Evol..

[62] Singh D.K., Sharma V.K., Kumar J., Mishra A., Verma S.K., Sieber T.N., Kharwar R.N. (2017). Diversity of endophytic mycobiota of tropical tree *Tectona grandis* Linn.f.: Spatiotemporal and tissue type effects. Sci. Rep..

[63] Tan X.-M., Zhou Y.-Q., Zhou X.-L., Xia X.-H., Wei Y., He L.-L., Tang H.-Z., Yu L.-Y. (2018). Diversity and bioactive potential of culturable fungal endophytes of *Dysosma versipellis*; a rare medicinal plant endemic to China. Sci. Rep..

[64] Guo L.D., Hyde K.D., Liew E.C.Y. (2000). Identification of endophytic fungi from *Livistona chinensis* based on morphology and rDNA sequences. New Phytol..

[65] Wang Y., Guo L.D., Hyde K.D. (2005). Taxonomic placement of sterile morphotypes of endophytic fungi from *Pinus tabulaeformis* (Pinaceae) in northeast China based on rDNA sequences. Fungal Divers..

[66] Sun X., Guo L.D. (2012). Endophytic fungal diversity: Review of traditional and molecular techniques. Mycology.

[67] Sandoval-Denis M., Crous P.W. (2018). Removing chaos from confusion: Assigning names to common human and animal pathogens in *Neocosmospora*. Persoonia.

[68] Diogo H.C., Sarpieri A., Pires M.C. (2005). Preservação de fungos em água destilada. Anais Bras. Dermatol..

[69] Burdsall H.H., Dorworth E.B. (2007). Preserving cultures of wood-decaying Basidiomycotina using sterile distilled water in cryovials. Mycologia.

[70] Okafor N. (2007). Modern Industrial Microbiolohy and Biotechnology.

[71] Sarma P., Dkhar M.S., Kayang H., Kumar M., Dubey N.K., Raghuwanshi R. (2018). Diversity of endophytic fungi associated with the medicinally important aromatic plant *Gaultheria fragrantissima*. Stud. Fungi.

[72] Selim K.A., Waill A.E., Ahmed M.T., Ahmed A.E.-B., Tahany M.A.-R., Ahmed I.E.-D., Eman F.A. (2018). Antiviral and antioxidant potential of fungal endophytes of Egyptian medicinal plants. Fermentation.

[73] Mahmoud A.G.Y., Zaher E.H.F. (2015). Why nuclear ribosomal Internal Transcribed Spacer (ITS) has been selected as the DNA barcode for fungi?. Adv. Gen. Eng..

[74] Chao A., Gotelli N.J., Hsieh T.C., Sander E.L., Ma K.H., Colwell R.K., Ellison A.M. (2014). Rarefaction and extrapolation with Hill numbers: A framework for sampling and estimation in species diversity studies. Ecol. Monogr..

[75] Alfenas A.C., Mafia R.G. (2016). Métodos em Fitopatologia.

[76] Carroll G. (1988). Fungal endophytes in stems and leaves: From latent pathogen to mutualistic symbiont. Ecology.

[77] Rajamanikyam M., Vadlapudi V., amanchy R., Upadhyayula S.M., Rajamanikyam M., Vadlapudi V., amanchy R., Upadhyayula S.M. (2017). Endophytic fungi as novel resources of natural therapeutics. Braz. Arch. Biol. Technol..

[78] Martin R., Gazis R., Skaltsas D., Chaverri P., Hibbett D. (2015). Unexpected diversity of basidiomycetous endophytes in sapwood and leaves of *Hevea*. Mycologia.

[79] Figueiredo Á., Silva A.C.e. (2014). Atividade in vitro de extratos de *Pycnoporus sanguineus* e *Lentinus crinitus* sobre o fitopatógeno *Fusarium* sp.. Acta Amazon..

[80] Iqbal M., Dubey M., Gudmundsson M., Viketoft M., Jensen D.F., Karlsson M. (2018). Comparative evolutionary histories of fungal proteases reveal gene gains in the mycoparasitic and nematode-parasitic fungus *Clonostachys rosea*. BMC Evol. Biol..

[81] Li J., Yang J., Huang X., Zhang K.Q. (2006). Purification and characterization of an extracellular serine protease from *Clonostachys rosea* and its potential as a pathogenic factor. Process Biochem..

[82] Zou C.G., Xu Y.F., Liu W.J., Zhou W., Tao N., Tu H.H., Huang X.W., Yang J.K., Zhang K.Q. (2010). Expression of a serine protease gene prCIs up-regulated by oxidative stress in the fungus *Clonostachys rosea*: Implications for fungal survival. PLoS ONE.

[83] Melo D.I.S., Valente A.M.M.P., Kavamura V.N., Vilela E.S.D., Faull J.L. (2014). Mycoparasitic nature of *Bionectria* sp. strain 6.21. J. Plant Protect. Res..

[84] Salamone A.L., Gundersen B., Inglis D.A. (2018). *Clonostachys rosea*, a potential biological control agent for *Rhizoctonia solani* AG-3 causing black scurf on potato. Biocontr. Sci. Technol..

[85] Vivas J.M.S., da Silveira S.F., dos Santos P.H.D., Carvalho B.M., Poltronieri T.P.D.S., Jorge T.S., Santos J.S., Kurosawa R.D.N.F., de Moraes R. (2018). Antagonism of fungi with biocontrol potential of papaya black spot caused by *Asperisporium caricae*. Austr. J. Crop Sci..

[86] Hassan M.M., Daffalla H.M., Modwi H.I., Osman M.G., Ahmed I.I., Gani M.E.A., El A., Babiker G.E. (2013). Effects of fungal strains on seeds germination of millet and *Striga hermonthica*. Univ. J. Agricult. Res..

[87] Hung R., Lee Rutgers S. (2016). Applications of *Aspergillus* in plant growth promotion. New Future Dev. Microbial Biotechnol. Bioeng..

[88] Pereira F.T., Oliveira D.J.B., Muniz P.H.P.C., Peixoto G.H.S., Guimarães R.R., Carvalho D.D.C., Pereira F.T., Oliveira D.J.B., Muniz P.H.P.C., Peixoto G.H.S. (2019). Growth promotion and productivity of lettuce using *Trichoderma* spp. commercial strains. Horticult. Bras..

[89] Waqas M., Khan A.L., Hamayun M., Shahzad R., Kang S.-M., Kim J.-G., Lee I.-J. (2015). Endophytic fungi promote plant growth and mitigate the adverse effects of stem rot: An example of *Penicillium citrinum* and *Aspergillus terreus*. J. Plant Interact..

[90] Xia C., Li N., Zhang X., Feng Y., Christensen M.J., Nan Z. (2016). An Epichloë endophyte improves photosynthetic ability and dry matter production of its host *Achnatherum inebrians* infected by *Blumeria graminis* under various soil water conditions. Fungal Ecol..

[91] Zavala-Gonzalez E.A., Rodríguez-Cazorla E., Escudero N., Aranda-Martinez A., Martínez-Laborda A., Ramírez-Lepe M., Vera A., Lopez-Llorca V.L. (2017). *Arabidopsis thaliana* root colonization by the nematophagous fungus *Pochonia chlamydosporia* is modulated by jasmonate signaling and leads to accelerated flowering and improved yield. New Phytol..

[92] Hardoim P.R., Van Overbeek L.S., Berg G., Pirttilä A.M., Compant S., Campisano A., Döring M., Sessitsch A. (2015). The hidden world within plants: Ecological and evolutionary considerations for defining functioning of microbial endophytes. Microbiol. Mol. Biol. Rev..

[93] Naraghi L., Heydari A., Rezaee S., Razavi M. (2012). Biocontrol agent *Talaromyces flavus* stimulates the growth of cotton and potato. J. Plant Growth Regul..

[94] Hossain M.M., Sultana F., Kubota M., Koyama H., Hyakumachi M. (2007). The plant growth-promoting fungus *Penicillium simplicissimum* GP17-2 induces resistance in *Arabidopsis thaliana* by activation of multiple defense signals. Plant Cell Physiol..

[95] Hamayun M., Afzal Khan S., Ahmad N., Tang D.-S., Kang S.-M., Na C.-I., Sohn E.-Y., Hwang Y.-H., Shin D.-H., Lee B.-H. (2009). *Cladosporium sphaerospermum* as a new plant growth-promoting endophyte from the roots of *Glycine max* (L.) Merr. World J. Microbiol. Biotechnol..

[96] Khalmuratova I., Kim H., Nam Y.-J., Oh Y., Jeong M.-J., Choi H.-R., You Y.-H., Choo Y.-S., Lee I.-J., Shin J.-H. (2015). Diversity and plant growth promoting capacity of endophytic fungi associated with halophytic plants from the west coast of Korea. Mycobiology.

[97] Priyadharsini P., Muthukumar T. (2017). The root endophytic fungus *Curvularia geniculata* from *Parthenium hysterophorus* roots improves plant growth through phosphate solubilization and phytohormone production. Fungal Ecol..

[98] Chithra S., Jasim B., Mathew J., Radhakrishnan E.K. (2017). Endophytic *Phomopsis* sp. colonization in *Oryza sativa* was found to result in plant growth promotion and piperine production. Physiol. Plant..

[99] Berlanas C., Berbegal M., Elena G., Laidani M., Cibriain J.F., Sagües A., Gramaje D. (2019). The fungal and bacterial rhizosphere microbiome associated with grapevine rootstock genotypes in mature and young vineyards. Front. Microbiol..

[100] Toju H., Okayasu K., Notaguchi M. (2019). Leaf-associated microbiomes of grafted tomato plants. Sci. Rep..

[101] De Silva D.D., Crous P.W., Ades P.K., Hyde K.D., Taylor P.W.J. (2017). Life styles of *Colletotrichum* species and implications for plant biosecurity. Fungal Biol. Rev..

[102] Fesel P.H., Zuccaro A. (2016). Dissecting endophytic lifestyle along the parasitism/mutualism continuum in *Arabidopsis*. Curr. Opin. Microbiol..

[103] O’Connell R.J., Thon M.R., Hacquard S., Amyotte S.G., Kleemann J., Torres M.F., Damm U., Buiate E.A., Epstein L., Alkan N. (2012). Lifestyle transitions in plant pathogenic *Colletotrichum* fungi deciphered by genome and transcriptome analyses. Nat. Genet..

[104] Corrêa R.C.G., Rhoden S.A., Mota T.R., Azevedo J.L., Pamphile J.A., de Souza C.G.M., Polizeli M.D.L.T.D.M., Bracht A., Peralta R.M. (2014). Endophytic fungi: Expanding the arsenal of industrial enzyme producers. J. Ind. Microbiol. Biotechnol..

[105] Toghueo R.M.K., Zabalgogeazcoa I., Vázquez de Aldana B.R., Boyom F.F. (2017). Enzymatic activity of endophytic fungi from the medicinal plants *Terminalia catappa*, *Terminalia mantaly* and *Cananga odorata*. S. Afr. J. Bot..

[106] Eken C., Demirci E. (2007). First report of *Colletotrichum truncatum* on alfalfa in Turkey. Plant Dis..

[107] Yang H.C., Haudenshield J.S., Hartman G.L. (2012). First report of *Colletotrichum chlorophyti* causing soybean anthracnose. Plant Dis..

[108] Górzyńska K., Węgrzyn E., Sandecki R., Lembicz M. (2019). Endophytic fungi and latent pathogens in the sedge *Carex secalina* (Cyperaceae), a critically endangered species in Europe. Plant Protect. Sci..

[109] Photita W., Lumyong S., Lumyong P., McKenzie E.H.C., Hyde K.D., Photita W., Lumyong S., Lumyong P., Hyde M.E.H.C. (2004). Are some endophytes of *Musa acuminata* latent pathogens?. Fungal Divers..

[110] Sessa L., Abreo E., Lupo S. (2018). Diversity of fungal latent pathogens and true endophytes associated with fruit trees in Uruguay. J. Phytopathol..

[111] Slippers B., Wingfield M.J. (2007). Botryosphaeriaceae as endophytes and latent pathogens of woody plants: Diversity, ecology and impact. Fungal Biol. Rev..

[112] Soumya P.R., Rukshana Begum S., Tamil Selvi K.S. (2018). Endophytic fungi as latent pathogens in *Eichhornia crassipes* (Mart.) Solms. Int. J. Adv. Sci. Res. Manag..

[113] Petrini O., Andrews J.H., Hirano S.S., Andrews J.H., Hirano S.S. (1991). Fungal endophytes of tree leaves. Microbial Ecology of Leaves.

[114] Verhoeff K. (1974). Latent infections by fungi. Ann. Rev. Phytopathol..

[115] Shade A., Jacques M.A., Barret M. (2017). Ecological patterns of seed microbiome diversity, transmission, and assembly. Curr. Opin. Microbiol..

[116] Shahzad R., Khan A.L., Bilal S., Asaf S., Lee I.-J. (2018). What is there in seeds? Vertically transmitted endophytic resources for sustainable improvement in plant growth. Front. Plant Sci..

[117] Sousa N.R. (2004). Variabilidade Genética e Estimativas de Parâmetros Genéticos em Germoplasma de Guaranazeiro.

[118] Freeman S., Rodriguez R.J. (1993). Genetic conversion of a fungal plant pathogen to a nonpathogenic, endophytic mutualist. Science.

[119] Lofgren L.A., LeBlanc N.R., Certano A.K., Nachtigall J., LaBine K.M., Riddle J., Broz K., Dong Y., Bethan B., Kafer C.W. (2018). *Fusarium graminearum*: Pathogen or endophyte of North American grasses?. New Phytol..

[120] Slippers B., Boissin E., Phillips A.J.L., Groenewald J.Z., Lombard L., Wingfield M.J., Postma A., Burgess T., Crous P.W. (2013). Phylogenetic lineages in the Botryosphaeriales: A systematic and evolutionary framework. Stud. Mycol..

[121] Unterseher M., Gazis R., Chaverri P., Guarniz C.F.G., Tenorio D.H.Z. (2013). Endophytic fungi from Peruvian highland and lowland habitats form distinctive and host plant-specific assemblages. Biodivers. Conserv..

[122] Ovaskainen O., Nokso-Koivisto J., Hottola J., Rajala T., Pennanen T., Ali-Kovero H., Miettinen O., Oinonen P., Auvinen P., Paulin L. (2010). Identifying wood-inhabiting fungi with 454 sequencing—What is the probability that BLAST gives the correct species?. Fungal Ecol..

[123] Tedersoo L., Nilsson R.H., Abarenkov K., Jairus T., Sadam A., Saar I., Bahram M., Bechem E., Chuyong G., Kõljalg U. (2010). 454 Pyrosequencing and Sanger sequencing of tropical mycorrhizal fungi provide similar results but reveal substantial methodological biases. New Phytol..

[124] Unterseher M., Schnittler M. (2009). Dilution-to-extinction cultivation of leaf-inhabiting endophytic fungi in beech (*Fagus sylvatica* L.)—Different cultivation techniques influence fungal biodiversity assessment. Mycol. Res..

[125] Zhang T., Yao Y.-F. (2015). Endophytic fungal communities associated with vascular plants in the high Arctic zone are highly diverse and host-plant specific. PLoS ONE.

[126] Christian N., Sullivan C., Visser N.D., Clay K. (2016). Plant host and geographic location drive endophyte community composition in the face of perturbation. Microbial Ecol..

[127] Hoffman M.T., Arnold A.E. (2008). Geographic locality and host identity shape fungal endophyte communities in cupressaceous trees. Mycol Res..

[128] David A.S., Seabloom E.W., May G. (2016). Plant host species and geographic distance affect the structure of aboveground fungal symbiont communities, and environmental filtering affects belowground communities in a coastal dune ecosystem. Microbial Ecol..

[129] De Arruda M.R., Clério J., Pereira R., Moreira A., Geraldes Teixeira W. (2007). Survival rate of guarana herbaceous cuttings in different substrates. Cienc. Agrotecnol..

[130] Garcia T.B., Nascimento Filho D.F.J. (1999). O Cultivo do Guarana no Amazonas.

[131] Albertino S.M.F., Filho F.J.D.N., da Silva J.F., Atroch A.L., Galvão A.K.D.L. (2012). Enraizamento de estacas de cultivares de guaranazeiro com adubação de plantas matrizes. Pesqui. Agropecu. Bras..

[132] Plácido C.G., Moreira A., Moraes L.A.C. (2015). Spacing and plant density in the yield components, nutritional status, and soil fertility of guarana varieties grown in humid tropical Amazon. Commun. Soil Sci. Plant Anal..

[133] Rosenberg E., Zilber-Rosenberg I. (2018). The hologenome concept of evolution after 10 years. Microbiome.

